# Polycarbonate/Poly(vinylidene fluoride)-Blend-Based Nanocomposites—Effect of Adding Different Carbon Nanofillers/Organoclay

**DOI:** 10.3390/polym13162626

**Published:** 2021-08-06

**Authors:** Fang-Chyou Chiu, Kartik Behera, He-Jie Cai, Yen-Hsiang Chang

**Affiliations:** 1 Department of Chemical and Materials Engineering, Chang Gung University, Taoyuan 333, Taiwan; b.kartik1991@gmail.com (K.B.); mkumari.biotech@gmail.com (H.-J.C.); 2 Department of General Dentistry, Chang Gung Memorial Hospital, Taoyuan 333, Taiwan; 3 Graduate Institute of Dental and Craniofacial Science, Chang Gung University, Taoyuan 333, Taiwan

**Keywords:** polycarbonate, poly(vinylidene fluoride), blend, nanocomposite, selective localization, physical properties

## Abstract

Carbon black (CB), carbon nanotubes (CNTs), and graphene nanoplatelets (GnPs) individually or doubly served as reinforcing fillers in polycarbonate (PC)/poly(vinylidene fluoride) (PVDF)-blend (designated CF)-based nanocomposites. Additionally, organo-montmorillonite (15A) was incorporated simultaneously with the individual carbon fillers to form hybrid filler nanocomposites. Microscopic images confirmed the selective localization of carbon fillers, mainly in the continuous PC phase, while 15A located in the PVDF domains. Differential scanning calorimetry results showed that blending PVDF with PC or forming single/double carbon filler composites resulted in lower PVDF crystallization temperature during cooling. However, PVDF crystallization was promoted by the inclusion of 15A, and the growth of β-form crystals was induced. The rigidity of the CF blend increased after the formation of nanocomposites. Among the three individually added carbon fillers, GnPs improved the CF moduli the most; the simultaneous loading of CNT/GnP resulted in the highest moduli by up to 33%/46% increases in tensile/flexural moduli, respectively, compared with those of the CF blend. Rheological viscosity results showed that adding CNTs increased the complex viscosity of the blend to a greater extent than did adding CB or GnPs, and the viscosity further increased after adding 15A. The electrical resistivity of the blend decreased with the inclusion of carbon fillers, particularly with CNT loading.

## 1. Introduction

Polymer blends and composites are fabricated to improve the specific properties of parent polymer components for broadening their applications. Blend/composite products have continuously attracted great attention from academia and industry. The miscibility, phase morphology, and type/dispersibility of added fillers are the key factors in controlling the final properties of polymer blends and composites. Polymer-based nanocomposites have received more interest than conventional polymer blends/composites in the last two decades [1,2]. The physical properties of polymer matrices can be drastically modified with a small amount of nanofiller. Research of different nanofiller(s) polymer nanocomposites continues to be updated [3,4,5,6]. Among the nanofillers used, organically modified montmorillonite (O-MMT, nanoclay) is one of the suitable choices for fabricating high-performance polymer nanocomposites. The most successful case is the well-known nylon 6/O-MMT nanocomposite system [7]. In addition to O-MMT, carbon nanomaterials with superior properties have also been utilized to manufacture polymer nanocomposites with enhanced properties. Carbon nanotubes (CNTs) and graphene nanoplatelets (GnPs) have demonstrated their potential to produce polymer nanocomposites possessing high rigidity and improved thermal/electrical conductivity [8,9,10,11]. It is recognized that a fine dispersion of nanofiller(s) throughout the polymer matrix and a strong attractive force between nanofiller(s) and a polymer are the two keys to successfully achieving polymer nanocomposites.

Bisphenol A-type polycarbonate (PC) is a well-known engineering plastic. It has diverse applications in the car, medicine, and electronics industries due to its high glass transition temperature, superior mechanical properties, transparency, and flame retardancy. PC-based nanocomposites have been prepared and studied. Hsieh et al. [12] successfully fabricated PC/O-MMT nanocomposites with enhanced rigidity. Their rheological study showed that the relaxation behavior of a PC matrix changed from liquid-like to pseudo-solid-like as the O-MMT content increased. Potschke et al. [13] reported that CNTs were finely dispersed in the PC matrix of masterbatch-prepared PC/CNT nanocomposites. The electrical percolation of the nanocomposites was determined to be between 1 and 1.5 wt.% CNT loading. Lai et al. [14] fabricated CNT-added PC nanocomposites and examined the effect of adding CNTs on the foaming properties of the PC matrix. The composites showed superior thermal stability and an improved storage modulus compared with neat PC; a rheological percolation threshold was determined at 2 wt.% CNT loading. Mohammadi et al. [15] studied PC/O-MMT and PC/graphene oxide (GO) nanocomposites. Both composite systems exhibited improved elastic and flexural moduli. The best loadings of the levels considered for PC/O-MMT and PC/GO nanocomposite systems were 1 and 0.6 wt.%, respectively.

Poly(vinylidene fluoride) (PVDF) is an important engineering plastic possessing unique dielectric properties, excellent thermal/chemical stability, and other advantageous properties [16]. Among its five distinct crystalline polymorphs (α, β, γ, δ, and ε), the nonpolar α form is the most stable and frequently encountered form. The polar β and γ forms offer piezo- and pyroelectric characteristics, allowing for advanced PVDF applications in electronic devices [17,18]. Priya and Jog [19] investigated melt-intercalated PVDF/O-MMT nanocomposites that exhibited an improved storage modulus compared with a neat PVDF matrix. Good piezoelectric property and increased ductility were achieved in PVDF/O-MMT nanocomposites reported by Yang et al. [20]. A high content of polar β-form crystals and a highly aligned PVDF structure were developed in the composites by applying shear force. Chiu [21] prepared and compared the properties of PVDF/O-MMT and PVDF/CNT nanocomposites. CNTs were found to exhibit better nucleation efficiency than O-MMT in the crystallization of PVDF. The addition of CNTs hardly changed the PVDF crystal form, whereas O-MMT addition induced β-form PVDF crystal formation. Anand et al. [22] prepared and examined flexible PVDF/reduced graphene oxide (RGO) nanocomposite films. Adding RGO increased the content of β-form PVDF crystals, and the ferroelectric behavior of PVDF nanocomposite films showed improvement in remnant polarization values as compared with pure PVDF.

Hitherto, various combinations of polymer/nanofiller binary nanocomposite systems have been successfully fabricated and characterized. Recently, polymer-blend-based multicomponent nanocomposites have attracted great attention due to their potential in advanced applications [23,24,25,26,27,28,29,30,31]. PC-blend-based and PVDF-blend-based nanocomposites have been studied to extend their individual versatility. Chiu et al. [23,24,25] investigated miscible PVDF/poly(methyl methacrylate)- and PVDF/poly(vinyl acetate)-blend-based nanocomposites with O-MMT, CNTs, and GnPs serving as nanofillers. The dispersibility of nanofillers and the crystallization/melting behavior of PVDF in the nanocomposites were evaluated. The rigidity of parent blends was evidently enhanced after adding the various nanofillers. Cao et al. [26] studied PVDF/polystyrene (PS)-blend-based nanocomposites with the incorporation of CNTs and silicon carbide (SiC). Selective localization of hybrid fillers (CNT/SiC) in PVDF domains led to high thermal conductivity and high electrical resistivity of the nanocomposite prepared. Li et al. [27] fabricated PVDF/polycaprolactone (PCL)/CNT nanocomposites in which the CNTs were localized in the PCL phase, resulting in a desirable double percolation structure. The nanocomposites exhibited much higher electrical conductivity than PVDF or PCL composites at the same CNT loading. Urtekin and Aytac [28] investigated the addition of functionalized CNTs on the properties of a PC/poly(lactic acid) (PLA) blend. The thermal stability, tensile properties, and electrical conductivity of the blend were improved to different degrees after adding differently functionalized CNTs.

According to the literature, nanocomposites fabricated with a combination of polymer blends and hybrid nanofillers have seldom been investigated. To broaden the applications of polymer blends, the individual and simultaneous loading of diverse nanofillers on the resultant properties is worthy of systematic investigation. In a previous study, PVDF/PC-70/30-blend-based nanocomposites were prepared and characterized [32]. In the PVDF-dominated blend matrix, the added carbon fillers were localized in the dispersed PC domains. The addition of CNTs led to the development of a quasi-co-continuous PVDF-PC morphology. The CNT/O-MMT hybrid filler drastically increased the rigidity of the parent blend. Even if some significant findings were revealed in a previous study, the properties of a PC-dominated blend and nanocomposites are still lacking of understanding. Therefore, the PC/PVDF-blend-nanocomposite system with PC as a matrix merits further examination. In the current study, continuing a previous study, the effect of the individual incorporation of three different carbon nanofillers on the physical properties of a PC-dominated PC/PVDF-70/30 blend was assessed. The further incorporation of O-MMT (hybrid fillers) in the carbon nanofiller composites on the final properties was also investigated. The dispersibility of nanofiller(s) in the PC/PVDF blend, and the thermal, mechanical, and rheological properties of the nanocomposites were characterized and compared. A decrease in the electrical resistivity of the parent blend after adding carbon nanofiller(s) was also highlighted.

## 2. Materials and Methods

### 2.1. Materials and Sample Preparation

Bisphenol A-type PC (Tarflon IR 1700, Taiwan Chemical Fiber Co., Taipei, Taiwan) with a melt flow rate of 30 g/10 min was used as the main component for preparing samples. PVDF (Sigma-Aldrich, weight average molecular weight: 180,000 g/mol) was used to blend with PC to form a PC-based blend. Commercially available carbon nanofillers, multiwalled CNTs, GnPs, and carbon black (CB), were used for the preparation of composites. The CNTs (Golden Innovation Co., Hsinchu, Taiwan) had a carbon purity of >99% and an average outer diameter of 60 nm. The GnPs (xGnP M-15, XG Sciences, Lansing, MI, USA) possessed high purity (>99.5%) and a thickness of about 6–8 nm. The CB (Vulcan XC-72, Cabot, Billerica, MA, USA) had an average diameter of 30 nm. In addition to the three different carbon nanofillers, commercial O-MMT (Cloisite 15A, denoted as 15A, Southern Clay Products Inc., Gonzales, TX, USA) was used as the extra nanofiller for the preparation of hybrid filler composites. Cloisite 15A is reported to have been modified with ca. 40% of dimethyl dihydrogenated tallow quaternary ammonium ion, and the tallow is composed of ca. 65% C18, 30% C16, and 5% C14. The cation exchange capacity of 15A is 125 mequiv/100 g.

PC/PVDF-blend and blend-based composites were melt-mixed using a Haake PolyDrive internal mixer (R600) with a Banbury-type rotor running at 60 rpm. Prior to mixing, the components were dried in an oven (Mandarin Scientific Co. Ltd., model: RUD-30 L, Taipei, Taiwan) at 70 °C for 24 h to remove absorbed moisture. The blend and composites were designed to have a PC-PVDF weight ratio of 7 to 3. The components (total 56 g) were mixed at 240 °C for 13 min. PC and PVDF components were first mixed for 5 min, and then the nanofiller(s) was incorporated with mixing continued for 8 min. The sample code of CF denotes the PC/PVDF blend, and CF-B, CF-T, CF-P, CF-BA, CF-TA, CF-PA, CF-TP, CF-TB, and CF-BP represent 1 wt.% of the different nanofillers loaded into the blend. B, T, P, and A denote CB, CNTs, GnPs, and 15A, respectively. For example, CF-T is the composite with 1 wt.% CNT, and CF-PA is the composite with 1 wt.% each of GnPs and 15A. For comparison, neat PC and PVDF were melt-treated under the same mixing conditions.

### 2.2. Characterization

Phase morphology and nanofiller(s) dispersion of the samples were assessed by a scanning electron microscope (SEM) combined with an energy dispersive spectroscope (EDS, Hitachi High-Technologies Corp., Tokyo, Japan). The compression-molded samples were fractured after immersion in liquid nitrogen and coated with gold prior to observation. A Hitachi S-3000N SEM (Hitachi High-Technologies Corp., Tokyo, Japan) was employed to conduct the experiments, and an “ImageJ” software was used to determine the domain size of the dispersed phase in the fractured blend/composites. An X-ray unit (XRD, D2 Phaser Bruker, Karlsruhe, Germany) with CuKα radiation (λ = 1.54 Å) was used to examine the PVDF crystal structure of the samples. The XRD was operated at 30 kV and 20 mA, and the compression-molded film samples were tested with a 2θ scan of 1.2°/min and a step size of 0.02°. A TA DSC Q10 (TA DSC Q10, New Castle, DE, USA) analyzer was employed to assess the crystallization and melting behavior of PVDF in different samples. For the crystallization study, samples of ca. 5 mg were first melted at 240 °C and then cooled to 20 °C at different rates. The precooled samples were afterwards scanned at 20 °C/min to 240 °C to evaluate the melting of PVDF. A thermogravimetric analyzer (TGA, TA Q50, TAinstrument, New Castle, DE, USA) was employed to study the thermal degradation behavior of the samples (ca. 10 mg). The samples were heated from room temperature to 680 °C at 20 °C/min in a nitrogen environment. The tensile modulus (TM) and flexural modulus (FM) of specimens prepared by compression molding (according to ASTMD638/ASTMD790) were determined at a strain rate of 2 mm/min and 1 mm/min, respectively, with a Gotech Al-3000 system (Taichung, Taiwan). The reported TM/FM values of each formulation are averages of five specimens. The storage modulus (SM) of compression-molded specimens (30 mm × 6 mm × 0.4 mm) was measured in N_2_ using a TA DMA Q800 (TA instrument, New Castle, DE, USA) system with measurements conducted in single cantilever mode over the temperature range of 25–150 °C at a 2 °C/min heating rate and a frequency of 1 Hz. An Anton Paar Physica rheometer (MCR 101; Anton Paar GmbH, Graz, Austria) in oscillating mode at 1% strain amplitude was utilized to assess the complex viscosity of the samples at 240 °C. The experiments were conducted with a parallel plate geometry (25 mm diameter and 1 mm gap), and the sweep frequency was in the range of 0.1–100 rad/s. The surface electrical resistivity of compression-molded specimens was measured using MCP-HT450 resistivity meters (Mitsubishi Chemical Co., Yamato, Japan). The reported values are the average of five repeated measurements obtained at various locations of each prepared strip.

## 3. Results and Discussion

### 3.1. Phase Morphology and Selective Localization of Nanofillers

**Figure 1:**
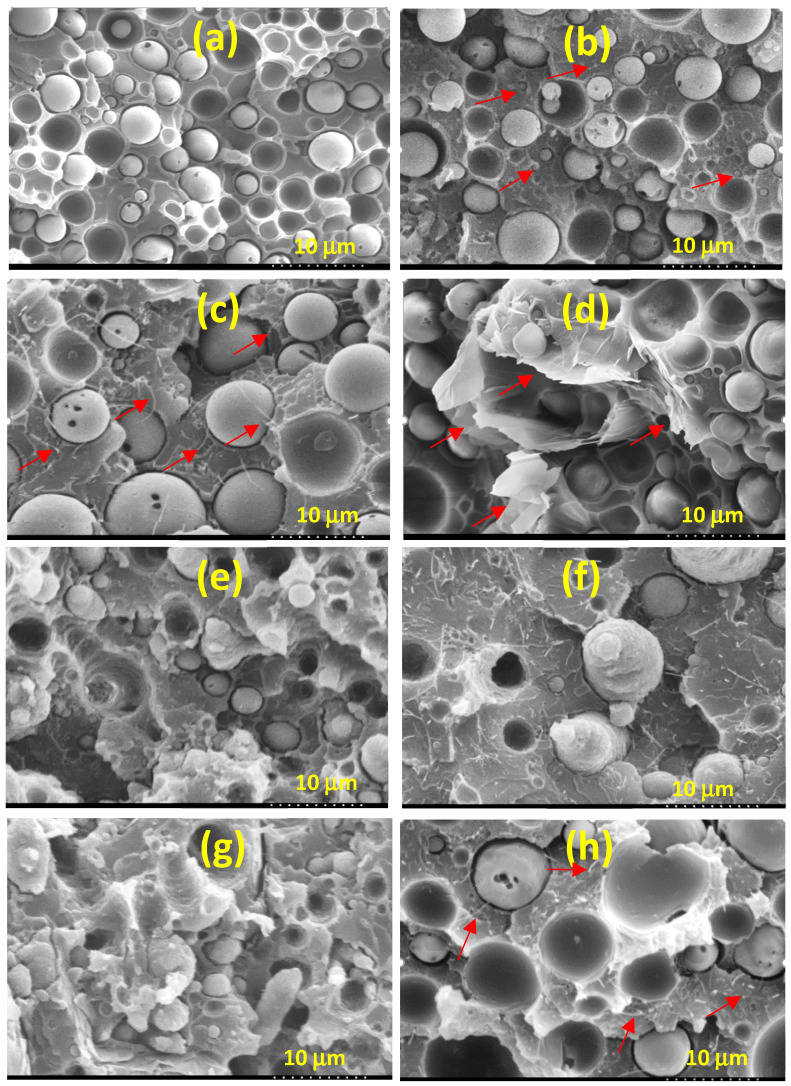
SEM images of: (**a**) CF (4.3 μm), (**b**) CF-B (6.9 μm), (**c**) CF-T (7.2 μm), (**d**) CF-P (6.8 μm), (**e**) CF-BA (5.5 μm), (**f**) CF-TA (5.8 μm), (**g**) CF-PA (5.6 μm), and (**h**) CF-TB (7.6 μm).

**Figure 2:**
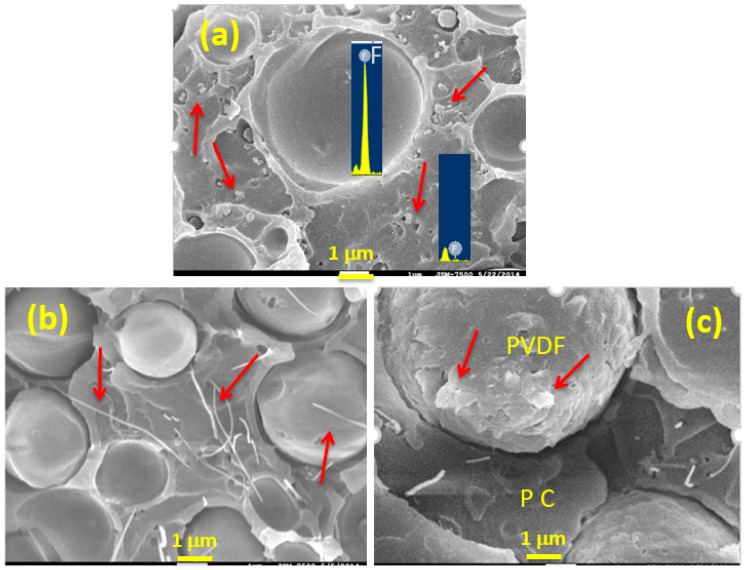
Higher-magnification SEM images of: (**a**) CF-B (with EDS data), (**b**) CF-T, and (**c**) CF-TA.

Figure 1 shows the SEM images of representative samples. As shown in Figure 1a, the CF blend exhibited sea–island morphology with the dispersed domains having an average size of about 4.3 μm. The identity of the dispersed domains was confirmed to be the minor component PVDF (results shown in Figure 2). Figure 1b–d shows the biphasic morphology after the addition of, respectively, carbon nanofillers CB, CNTs, and GNPs. The average size of the dispersed PVDF domains increased to above 6.8 μm with a larger size distribution in the composites. The average domain size (DS) is given in the caption of Figure 1. The SEM images also show that the carbon nanofillers (arrowed) were mostly located in the continuous PC phase. Some nanofiller was also observed at the interfacial regions of PC-PVDF phases. The increase in PVDF domain size was mainly attributed to the viscosity increase in the nanofiller-located PC phase, which resulted in an increased viscosity ratio between the PC and PVDF phases (see the following viscosity result). Figure 1e–g shows the images of composites with the coexistence of 15A and an individual carbon nanofiller (hybrid fillers). The DS of PVDF became smaller, evidently coarser, and irregularly shaped compared with that of the corresponding carbon nanofiller only samples. The alteration in the texture of PVDF domains was ascribed to the selective localization of 15A within the domains (more evident in Figure 2). The individual carbon nanofiller was again noted to be situated in the PC phase. Figure 1h illustrates a typical image (CF-TB) of two carbon nanofiller composites. Both fillers (arrowed) were located in the PC phase, and the PVDF domain size increased due to the increased viscosity difference between the two phases.

To clearly reveal the localization of added nanofiller(s) in the blend matrix, Figure 2 shows magnified SEM images of representative composites. Figure 2a shows an image of a CF-B composite along with EDS data. The CB (arrowed) was distributed finely in the continuous phase (matrix), but not detectable in the dispersed domains. As revealed by EDS, the dispersed domains showed an evident fluorine (F) signal, whereas F was hardly detected in the matrix. A similar EDS observation was made in the blend and other composite samples (not shown for brevity). The SEM/EDS results confirmed that the matrix was formed by the major component, PC, while the dispersed domains were composed of the PVDF component. Figure 2b shows that CNTs (arrowed) were mainly localized in the PC phase of CF-T, with some CNTs on the surface of the PVDF domains. The image in Figure 2c confirms the localization of CNTs and 15A in the respective PC and PVDF phases. The layered 15A is indicated with arrows. The above morphological observations indicate that 15A had more affinity for PVDF than for PC, while the carbon nanofillers preferred to localize in the PC phase (driven mainly by thermodynamics [27]).

### 3.2. Crystal Structure

**Figure 3:**
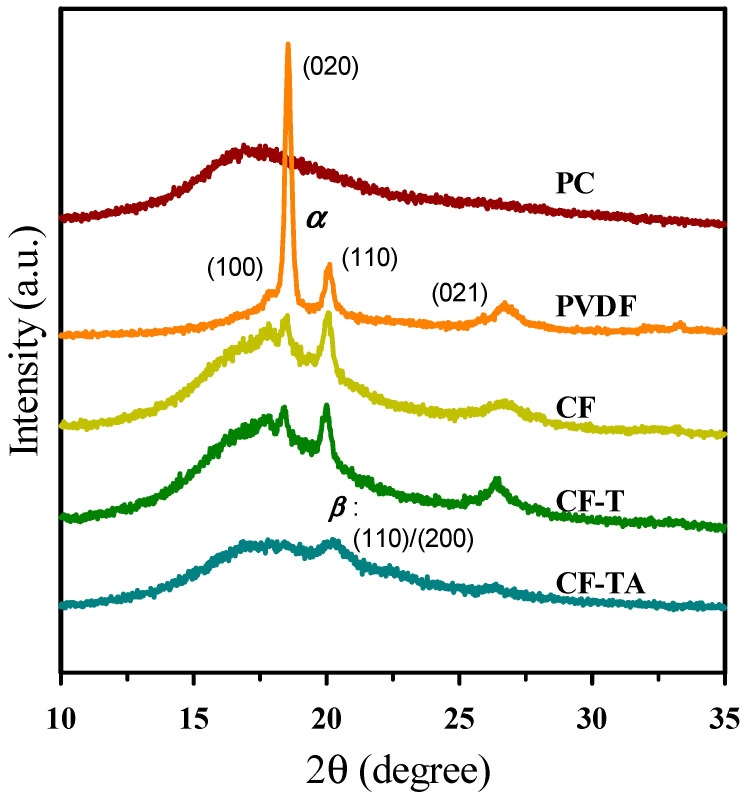
XRD patterns of 5 °C/min cooled representative samples.

XRD patterns of neat components and the CF blend cooled at 5 °C/min from the melt state are illustrated in Figure 3. PC displayed a typical amorphous halo in the 2θ range of ca. 15° to 22°. Neat PVDF exhibited a stable α-form, with diffractions located at 2θ = 17.9° (100), 18.5° (020), 20.0° (110), and 26.6° (021). For the CF blend, the intensity of PVDF diffractions drastically reduced because of the large portion of the amorphous PC component. The composite having only the addition of a carbon nanofiller (cf. CF-T) showed the α-form diffractions of PVDF, similar to the pattern of the CF blend. CF-B and CF-P also exhibited PVDF α-form diffractions (data not shown for brevity). For the representative 15A-included composite (CF-TA), the diffraction (e.g., (100)) intensities of PVDF α-form crystals were much diminished. A broad diffraction appeared at 2θ = 20.6°, which is associated with the β-form ((110)/(200)) crystals. The modification in the XRD patterns demonstrates the induction of polar β-form PVDF crystals after the addition of 15A, in agreement with previous reports [19,20,21,32]. The result corresponds well to the morphological observation of 15A locating in the PVDF domains, and thereby had an influence on the PVDF polymorph.

### 3.3. Crystallization and Melting Behavior

**Figure 4:**
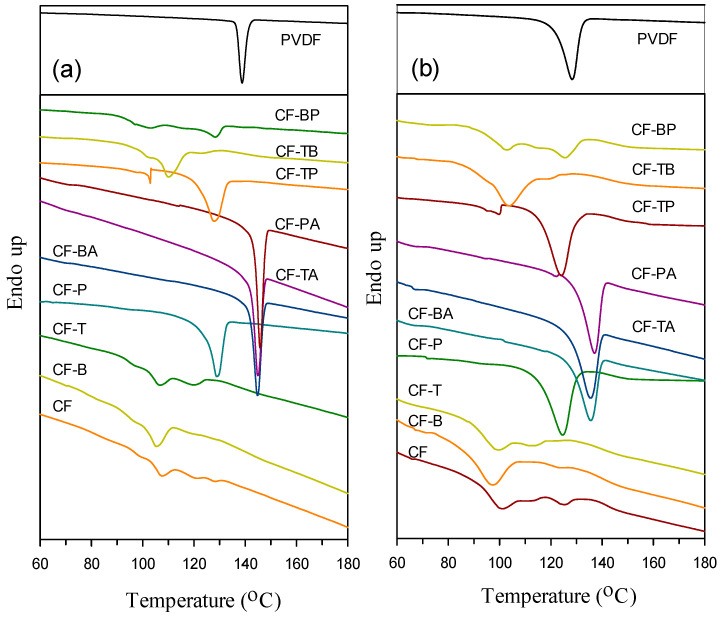
DSC cooling curves of (**a**) 5 °C/min and (**b**) 40 °C/min cooled samples.

The effects of blending with PC and adding different nanofillers on the crystallization of PVDF were investigated through DSC. Figure 4a depicts the 5 °C/min cooling curves of the samples (from the melt state). Since the PVDF crystallization enthalpy declined drastically in the blend and composites, the enthalpy (y-axis) scales of neat PVDF and the blend/composites are plotted differently in the figure in order to compare the crystallization of PVDF in different samples. The crystallization peak temperature (T_p_, at the exotherm minimum) of neat PVDF was around 139 °C, and it shifted noticeably to a lower temperature (main peak) of ca. 107 °C after CF blend formation. The evident shift should be attributed to the homogeneous nucleation of PVDF in the various small PVDF domains in the blend [5]. The complicated exotherm (multiple peaks) of the blend was related to the distribution of different PVDF domain sizes (different degrees of homogeneous nucleation). The CF-B and CF-T composites displayed a similar crystallization behavior/temperature as the CF blend, because of their PVDF domain size being comparable to that of the CF blend. The selective localization of CB and CNTs in the PC phase leading to no effect on PVDF crystallization (nucleation) was also confirmed. However, the CF-P composite had a single exothermic peak with the T_p_ value (ca. 129 °C) evidently higher than that of the CF blend. This observation might be ascribed to the large dimensions of GnPs inducing less homogeneous nucleation in PVDF domains. That is, GnPs had somehow contacted the PVDF domains, and then led to a minor nucleation effect for PVDF. For the 15A-included (hybrid filler) composites, the crystallization of PVDF appeared at higher temperatures compared with that of neat PVDF. The higher-temperature PVDF crystallization could have stemmed from the 15A-nucleated/induced growth of α- and β-form crystals, as 15A was distributed in the PVDF domains. For the hybrid two carbon nanofiller composites, the GnP-added ones showed a main peak close to that of CF-P. The GnPs-accelerated PVDF crystallization in the composites was again demonstrated. The main T_p_ values of the samples are compared in Table 1. Figure 4b shows the 40 °C/min cooled curves of the samples. The crystallization behavior of individual samples is similar to those cooled at 5 °C/min. The slight decrease in crystallization temperatures for the 40 °C/min cooled samples compared with their 5 °C/min cooled counterparts was mainly due to the thermal lag effect [3,5].

**Figure 5:**
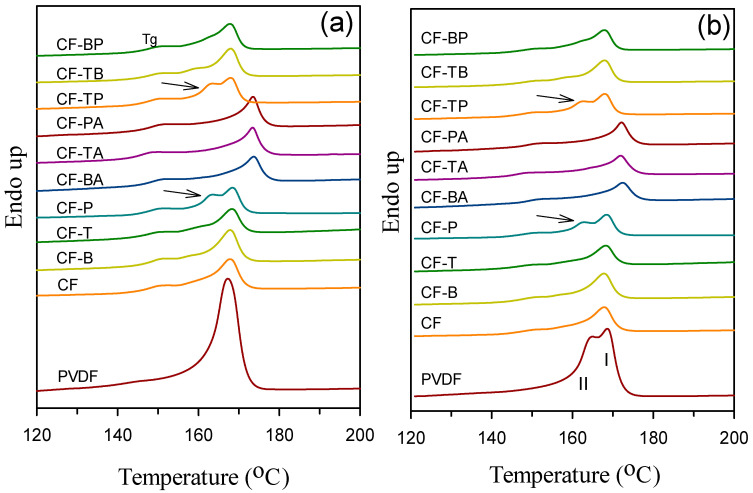
DSC heating curves of (**a**) 5 °C/min and (**b**) 40 °C/min precooled samples.

Figure 5a depicts the DSC melting endotherm of 5 °C/min cooled samples. The neat PVDF and samples without 15A inclusion displayed a main PVDF melting peak (T_m_) at 167–169 °C. The transition around 147–152 °C of each PC-included curve was the glass transition of PC. An evidently lower-temperature endotherm (arrowed) partially overlapping the main melting peak was additionally seen for CF-P and CF-TP samples. The two meltings of PVDF were associated with the individual melting of originally grown and heating-process-recrystallized *α*-form crystals. The evident lower temperature melting observed in CF-P/CF-TP suggests that GnPs must have caused the growth of more stable original crystals compared with CB and CNTs. As a consequence, the melting of original crystals could be observed along with the melting of heating-recrystallized, more stable crystals. That is, a less amount of crystals grew during the heating scan of CF-P/CF-TP. The relatively higher crystallization temperatures (T_p_ values) of CF-P/CF-TP (Figure 4) are responsible for the observation. For the 15A-included composites, a higher single T_m_ value (at around 173 °C) than that of neat PVDF was observed. Per XRD data, a higher T_m_ could be associated with the melting overlap of 15A-induced β-form crystals and some α-form crystals [32]. The main T_m_s of the samples are summarized in Table 1. Figure 5b depicts the melting behavior of the 40 °C/min precooled samples. Neat PVDF exhibited two partially overlapping peaks due to the melting of originally grown, less stable crystals (peak II) and the heating-recrystallized/annealed crystals (peak I). The corresponding 5 °C/min precooled PVDF grew very few of the less stable crystals, and hence exhibited the melting of originally grown stable crystals (single peak) only. The blend and composites showed a similar melting behavior to the 5 °C/min cooled counterparts with a smaller melting endotherm (lower crystallinity).

### 3.4. Thermal Stability

**Figure 6:**
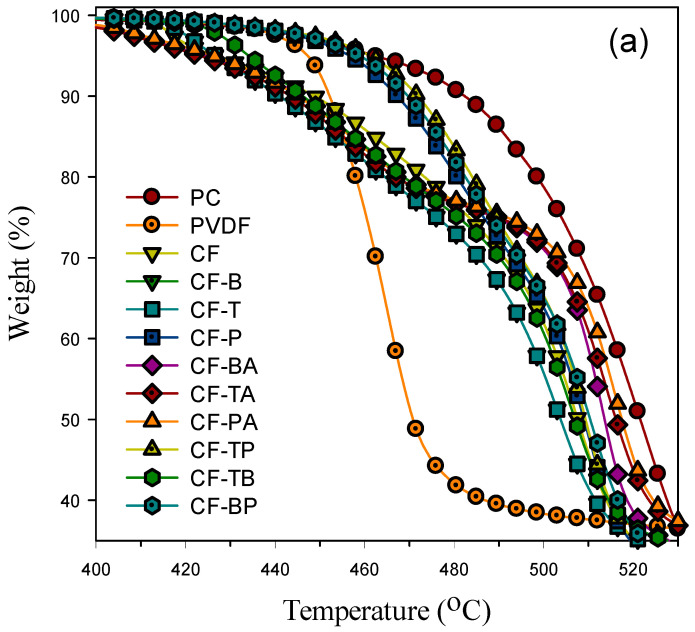

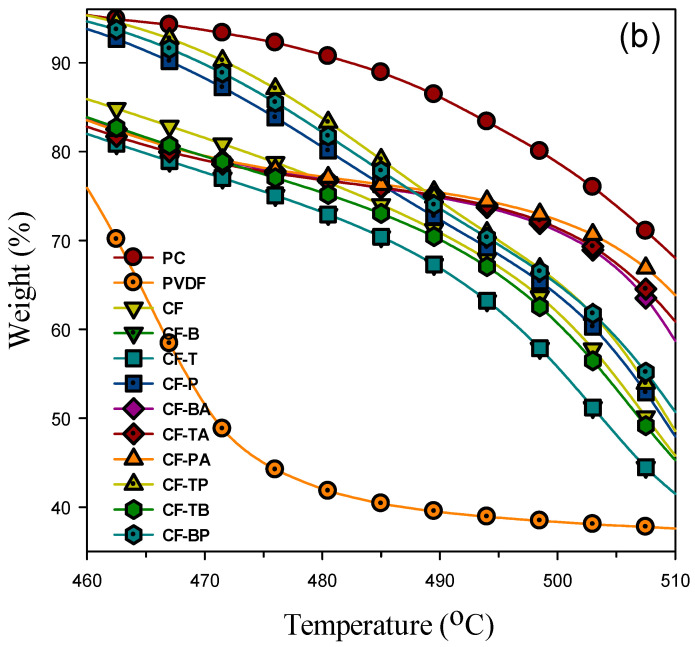
TGA curves of tested samples plotted under a different temperature range for (**a**,**b**).

Figure 6a shows the thermal degradation behavior (TGA curves) of the samples tested in a nitrogen environment. Neat PVDF started an evidently lower degradation temperature at around 447 °C (5% weight loss), and PC started its degradation at a higher temperature of 462 °C. The CF blend exhibited a degradation curve basically in between those of its parent PVDF and PC components. However, the PVDF degradation started at a lower temperature in the blend compared with that in the neat state. The existence of PC had caused a negative effect on the thermal degradation of PVDF, with the reason not yet clear. For the composites having only CB and/or CNTs included, the degradation was similar to that of the blend, with a slightly lower degradation temperature. The selective location of CB and CNTs in the PC major phase had no positive effect on the PVDF and PC degradation. The composites with GnP inclusion (CF-P/CF-TP/CF-BP, excluding CF-PA) displayed evidently higher degradation temperatures in the PVDF part, indicating the prevention of heat transfer and evaporation of degraded fragments by the large dimension of layered GnPs (surrounding the PVDF domains). The positive effect in thermal degradation was CF-TP > CF-BP > CF-P, as clearly shown in Figure 6b. Regarding the PC part, the effect of GnPs was much reduced. Figure 6b illustrates that adding 15A along with the carbon nanofillers (CF-BA/CF-TA/CF-PA) marginally affected the degradation in the PVDF part. On the other hand, the degradation temperature in the PC part was obviously increased. The observation reveals that the layered structure of 15A had the effect of indirectly enhancing the thermal stability for PC, but not for PVDF. The effects of the selective localization of different nanofillers and the large dimension of GnPs are responsible for the TGA observations. The temperatures at 5% (T_d5_, mainly PVDF degradation) and 50% (T_d50_, mainly PC degradation) weight loss for the individual samples are compared in Table 1.

### 3.5. Mechanical Properties

**Figure 7:**
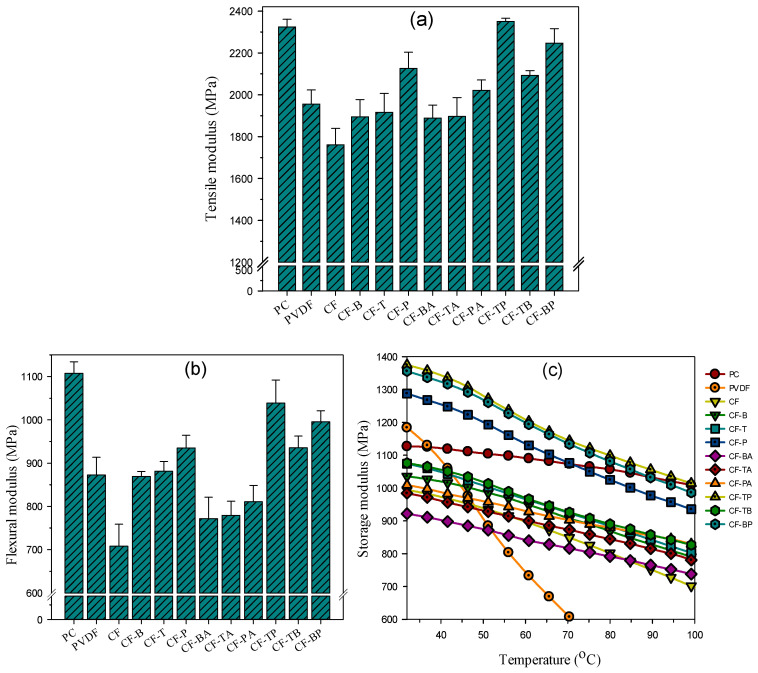
(**a**) Tensile modulus, (**b**) flexural modulus, and (**c**) storage modulus of prepared samples.

Figure 7 compares the formulation-dependent tensile modulus (TM)/flexural modulus (FM)/storage modulus (SM) of prepared samples. In Figure 7a, PC showed a higher TM value than that of PVDF. The blend, however, had a value lower than that of parent PC and PVDF due to its immiscible nature. The TM values of individual samples are listed in Table 1. As the carbon nanofillers were mainly localized in the continuous PC phase, an evident increase in TM was observed in the composites with the sole addition of a carbon nanofiller. Comparing TM reinforcement for the blend, GnPs exhibited superior efficiency to CB and CNTs, which could be ascribed to the larger dimension of its layered structure. The addition of GnPs (cf. CF-P) increased TM by approximately 21% compared with that of the blend. CNTs showed slightly higher reinforcing efficiency than CB because of their high aspect ratio. For the hybrid filler systems with 15A inclusion, the TM decreased slightly compared with the sole carbon nanofiller composites. The alteration in the biphasic morphology and the localization of 15A in PVDF domains are responsible for the observation. The 15A might have caused minor degradation of PVDF during the mixing procedure (TGA data). The double carbon filler composites exhibited higher TM compared with the single carbon filler systems, especially with GnP inclusion. The CF-TP exhibited the highest TM of the samples tested, with 33% increase compared with the CF blend. The FM of the samples exhibited a similar formulation-dependent trend to that of TM, as shown in Figure 7b. The CF blend had the lowest value, and the value increased for the composites. The FM values are also summarized in Table 1. GnPs again exhibited higher efficiency than CB/CNTs in enhancing the FM of the CF blend. The FM of CF-TP increased by 46% compared with that of the blend. The coexistence of 15A and the respective carbon nanofiller again caused a decline in FM. A further study is required to find the origin of TM/FM decline in composites that included 15A. Figure 7c shows the SM vs. temperature plots of the samples. As anticipated, SM decreased with increasing temperature, and PVDF was more sensitive to temperature (due to the α-transition relaxation in the crystalline region of the PVDF chains [33]) compared with the other samples in the test temperature region. The additional inclusion of 15A again decreased the SM of the corresponding single carbon filler composites. The effectiveness of carbon nanofillers in increasing SM followed the sequence GnP > CNT > CB. The CF-TP exhibited the highest SM at all temperatures tested.

### 3.6. Rheological and Electrical Properties

**Figure 8:**
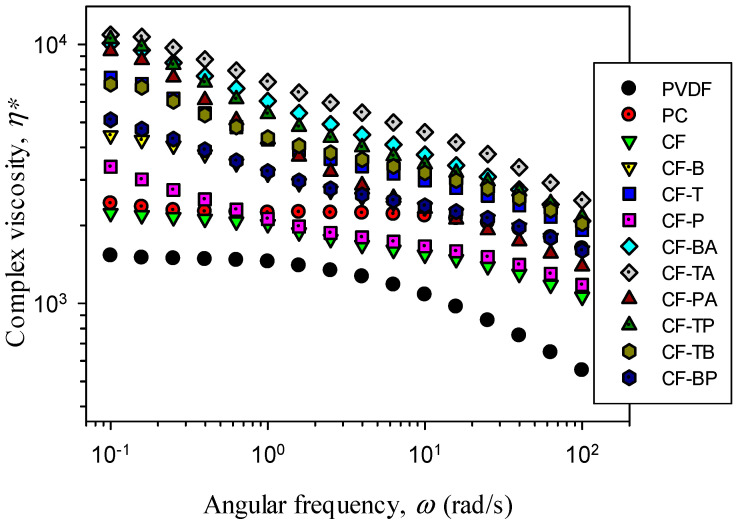
Complex viscosity (η*) vs. frequency (ω) of the samples.

Figure 8 illustrates the complex viscosity (η*) vs. frequency (ω) of the samples. Neat PC, PVDF, and the CF blend exhibited a Newtonian fluid behavior (flattened slope) at low frequencies; PC had a higher η* than PVDF at all frequencies. The η* values of the blend were in between those of PC and PVDF. For all the composites, higher η* values were observed at all frequencies compared with those of the CF blend. Furthermore, the composites exhibited a non-Newtonian fluid behavior starting from low frequencies, suggesting the formation of a pseudo-network structure (solid-like behavior) by the dispersed carbon nanofiller(s) in the PC major phase. The single carbon filler composites possessed lower η* values compared with the hybrid nanofiller systems. The increase in η* for the single carbon nanofiller system followed the sequence CNT > CB > GnP. The high aspect ratio and superior dispersion of CNTs caused the evident increase in η*, whereas the inferior dispersion of layered GnPs modified η* the least. The additional inclusion of 15A in the PVDF domains evidently increased η* for the single carbon filler composites, following the sequence TA > BA > PA. The evident increase might be caused by the double filler network development in the individual PC and PVDF phases. As anticipated, the double carbon filler composites showed higher η* values compared with the single carbon filler composites. CF-TP exhibited the highest values of the double carbon filler composites. The dispersed CNTs and GnPs must have achieved a closely connected network structure in the PC phase. The increase in η* for the composites demonstrated that the addition of different single and hybrid nanofillers led to different degrees of elasticity increase. The modification of the biphasic morphology and the well-dispersed nanofiller(s) notably influenced the rheological property of the samples.

The addition of conductive nanofiller(s) to the polymer matrix may lead to (semi)conductive products. The surface electrical resistivity (ER) of the samples was measured, and values are listed in Table 1. The neat components and blend had ER values higher than 10^13^ Ω-cm, indicating their electrically insulating nature. The individual addition of 1 wt.% CB (CF-B) and 1 wt.% CNTs (CF-T) reduced the resistivity by 3–4 orders of magnitude to about 10^10^ and 10^9^ Ω-cm, respectively. For the 1 wt.% GnP composite (CF-P), the ER dropped only about 2 orders, from 10^13^ to 10^11^ Ω-cm. The different degrees of drop in ER after adding various carbon nanofillers are mainly attributed to the combined effects of aspect ratio and dispersion of the individual nanofillers. The size- and dispersion-related surface area of individual carbon nanofillers also played a role in influencing the resultant property [34]. CNTs showed a larger aspect ratio and better dispersion in the continuous PC phase, resulting in the best efficiency in reducing the ER. GnPs, although having a high aspect ratio, had the worst dispersion (some stacked layered structure), and thus reduced the ER the least. Unexpectedly, the ER of the single carbon filler composites increased after the addition of 15A. The alteration in the biphasic morphology with the selectively localized carbon nanofiller/15A could play an important role in this respect. The double carbon filler composites exhibited lower resistivity than single carbon filler composites due to higher loading of carbon nanofillers. The CF-TB had the lowest ER value of about 10^8^ Ω-cm, only 1 order lower compared with CF-T. Based on the result, it is expected that the CF blend with 2 wt.% CNT inclusion will exhibit lower ER than CF-TB (identical amount of carbon nanofiller inclusion). The percolation behavior of the single and double carbon filler composites will be investigated in a future study.

## 4. Conclusions

The dispersion status of individually and simultaneously added nanofillers (CB, CNTs, GnPs, and 15A) in a PC/PVDF-70/30 blend was evaluated. The achievement of single and hybrid filler nanocomposites was confirmed. The carbon fillers were mainly localized in the major PC phase, while 15A located in the dispersed PVDF domains. Blending with PC caused an evidently lower crystallization temperature of PVDF (due to the homogeneous nucleation), whereas adding 15A promoted PVDF crystallization and induced the formation of higher melting temperature β-form PVDF crystals (XRD result) in the hybrid filler nanocomposites. The addition of CB and/or CNTs barely changed the crystallization and melting behavior of PVDF in the blend, while adding GnPs modified those properties. The positive effect of GnPs on the thermal stability of the PC/PVDF blend was demonstrated, whereas CB and CNTs hardly had any influence. Adding a layered 15A increased the degradation temperature of PC in the composites. The rigidity (tensile/flexural moduli) of the blend increased after adding carbon nanofillers, and GnPs exhibited higher efficiency than CB and CNTs. The effectiveness of carbon nanofillers in increasing the storage modulus also followed the sequence GnP > CNT > CB. A slight decrease in rigidity was noticed when 15A was added to the single carbon filler composites. The double carbon filler composites exhibited higher rigidity compared with the single carbon filler systems; the coexistence of CNTs and GnPs exhibited the highest rigidity among the samples tested. An increase in complex viscosity was observed for the nanocomposites, suggesting that a pseudo-network structure was developed. CNTs led to a more evident increase compared with CB and GnPs; further addition of 15A increased the viscosity. The reduction in the electrical resistivity of the PC/PVDF blend followed the sequence of adding CNT > CB > GnP. The addition of 1 wt.% CNTs reduced the electrical resistivity by 4 orders of magnitude; the coexistence of 1 wt.% individual CNTs and CB dropped the resistivity the most, up to 5 orders. The presence of 15A increased the electrical resistivity of the single carbon filler composites.

## Figures and Tables

**Table 1:** Representative physical properties of the samples.

Samples	Properties						
	T_p_ (°C)	T_m_ (°C)	T_d5_ (°C)	T_d50_ (°C)	TM * (MPa)	FM * (MPa)	ER (Ω-cm)
PVDF	138.8	167.2	447	471	1956 (67)	873 (41)	>10^13^
PC	-----	----	462	522	2325 (36)	1107 (26)	>10^13^
CF	106.8	167.9	426	508	1760 (78)	708 (51)	>10^13^
CF-B	106.2	168.1	427	504	1895 (82)	869 (11)	3.2 × 10^10^
CF-T	106.5	168.5	428	506	1916 (90)	881 (23	6.0 × 10^9^
CF-P	128.7	168.6	457	509	2126 (77)	935 (29)	1.2 × 10^11^
CF-BA	144.8	173.6	425	514	1889 (62)	771 (50)	>10^13^
CF-TA	144.9	173.4	423	516	1897 (89)	779 (33)	2.4 × 10^12^
CF-PA	145.8	173.5	426	517	2021 (49)	811 (38)	>10^13^
CF-TP	127.8	168.0	461	509	2351 (15)	1039 (53)	3.3 × 10^9^
CF-TB	110.1	167.9	434	507	2093 (23)	936 (27)	6.4 × 10^8^
CF-BP	128.3	167.7	459	510	2247 (69)	995 (26)	1.9 × 10^10^

* Standard deviation for TM and FM is included.

## Data Availability

Data sharing not applicable.
